# Impact of the 2015 El Niño-Southern Oscillation on the Abundance and Foraging Habits of Guadalupe Fur Seals and California Sea Lions from the San Benito Archipelago, Mexico

**DOI:** 10.1371/journal.pone.0155034

**Published:** 2016-05-12

**Authors:** Fernando R. Elorriaga-Verplancken, Gema E. Sierra-Rodríguez, Hiram Rosales-Nanduca, Karina Acevedo-Whitehouse, Julieta Sandoval-Sierra

**Affiliations:** 1 Centro Interdisciplinario de Ciencias Marinas, Instituto Politécnico Nacional (CICIMAR-IPN), Departamento de Pesquerías y Biología Marina, Ave. IPN s/n Col. Playa Palo de Santa Rita, 23096, La Paz, Baja California Sur, Mexico; 2 Universidad Autónoma de Baja California Sur, Departamento Académico de Biología Marina, Carretera al sur, km 5.5, Apartado Postal 19-B, 23080, La Paz, Baja California Sur, México; 3 Unidad de Microbiología Básica y Aplicada, Facultad de Ciencias Naturales, Universidad Autónoma de Querétaro, Av. de las Ciencias S/N, Juriquilla, 76230, Querétaro, Qro, México; 4 The Marine Mammal Center, 2000 Bunker Road, Sausalito, California, 94965, United States of America; Sonoma State University, UNITED STATES

## Abstract

The abundance of California sea lions (*Zalophus californianus*) (CSLs) and Guadalupe fur seals (*Arctocephalus philippii townsendi*) (GFSs) from the San Benito Archipelago (SBA) was determined through nine monthly surveys in 2014–2015. Assessment of their foraging habits was examined based on the isotopic analysis of pups (maternal indicators) (SIAR/SIBER-R). Environmental variability between 2014 and 2015 was also analyzed, in terms of sea surface temperature (SST) and chlorophyll (Chl-a) concentration. Both otariids reached their highest abundance in July of both years; however, relative to 2014, the 2015 survey showed a 59.7% decline in the total GFS abundance and a 42.9% decrease of GFS pups, while total CSL abundance decreased 52.0% and CSL pup presence decreased in 61.7%. All monthly surveys for both otariids showed a similar trend (>50% decrease in 2015). Compared to 2014, the 2015 GFSs isotopic niche was three times larger (2.0 in 2015, 0.6 in 2014) and the δ^13^C was significantly lower. CSLs also showed significantly lower δ^13^C and higher δ^15^N in 2015. Interannual segregation was greater for CSLs, and their pup body mass was also significantly lower during the 2015 breeding season (mean = 8.7 kg) than in the same season of 2014 (mean = 9.9 kg). The decrease in δ^13^C for both otariids reflected a more oceanic foraging; most likely associated with the decline in primary productivity in surrounding areas to the SBA, related to a higher SST caused by the 2015 ENSO, with a subsequent increase in foraging effort. These would explain the fewer observed individuals on land, especially pups, which showed diminished body condition (CSLs). This study highlights the importance of marine mammals as sentinel species that respond dynamically to changes in environment, providing valuable information on the effect of ENSO on pinnipeds in Mexican waters.

## Introduction

Two otariid species inhabit Mexico, the Guadalupe fur seal (*Arctocephalus philippii townsendi*) (GFS) and the California sea lion (*Zalophus californianus*) (CSL). The former was hunted to near extinction in the 19^th^ century; however, once the species received protected status in the 1950s, its population began to recover on Guadalupe Island (GI) [1] to a current abundance of 17,000–20,000 individuals [2]. Re-colonization of one of its former distribution sites, the San Benito Archipelago (SBA), has also been observed [3]. With just one well-established breeding colony (GI), Mexican law identifies the GFS as an endangered species [4]. The CSL has a significantly broader distribution and greater abundance, with a population of around 390,000 and rookeries that span from California islands to the west coast of Baja California and the Gulf of California in Mexico [5–7]. The CSL is resident on the SBA throughout the year, with a stable abundance in recent years [8,9]. In contrast, the GFS continues to recolonize the SBA, as evidenced by their continuous increase on these islands since 1997, when 256 individuals were recorded [3]; since then, the population has increased [10,11]. Some argue that the species’ recovery will depend more on extrinsic factors such as prey availability, rather than genetic factors [12].

Female GFSs from GI undertake foraging trips that range up to 444 ± 151 km [13]. Based on scat and stable isotope analyses of pup fur during 2013, GFSs from GI and those from SBA had different diets. The former primarily consumed (77.7%) jumbo squid (*Dosidicus gigas*) and hooked squid (*Onychoteuthis compacta*); while the SBA individuals preyed mainly (90.1%) on opalescent squid (*Doryteuthis opalescens*). The isotopic analysis confirmed this segregation as well as the tendency for individuals from both colonies to forage in oceanic areas [14]. In contrast, CSL foraging trips are more coastal, and the species includes more fish species in their diet [15,16]. Scat analysis has revealed that CSL from the SBA consume prey like the Pacific silverweed (*Argentina sialis*), Panama hake (*Merluccius angustimanus*), and rockfishes (*Sebastes* spp.) [17]. For both otariids, foraging strategies vary by site and sampling period [15,18]. Both GFSs and CSLs exploit regional resources as part of their foraging strategy, alternating between foraging at sea and nursing their pups on land [19,20].

Pinniped distribution depends on prey availability near the rookeries or haulouts, which in turn is influenced by sea surface temperature (SST) [15,18,21]. The increase in SST during past El Niño-Southern Oscillation (ENSO) events and other similar phenomena, have negatively affected numerous pinniped populations [21]. In the Northeastern Pacific, an unusually large (~2,000 km wide) mass of warm water named “The Blob” has prevailed in recent years; this phenomenon was first observed off the southern coast of Alaska in December 2013, extending to the western part of Baja California by mid-2014 [22,23]. Moreover, a strong ENSO has been present in the Pacific since spring 2015 [24]. These events likely play a role in both otariids’ foraging habits and thus their yearly abundance on islands like the SBA.

Stable isotope analysis (δ^13^C and δ^15^N) is a useful tool for evaluating foraging habits at different time scales [25]. A predator’s δ^13^C reflects their prey’s habitat due to dissolved CO_2_ concentration, phytoplankton growth rate and composition, and presence of macrophyte algae in coastal ecosystems, which enriches the ^13^C at the base of the food web [26–28]. δ^15^N is an indicator of trophic level and breadth because the accumulation from prey to predator occurs in increments that are relatively predictable. Values range from 3 to 5‰ for δ^15^N and from 0.5 to 2‰ for δ^13^C [25,29–31]. Spatial variation in δ^15^N and δ^13^C at the base of the food web is negatively correlated with latitude in the Northeast Pacific [32–33]. These isotopic differences are associated with areas of denitrification/minimum oxygen zones at low-middle latitudes, which increase the δ^15^N at the base of the food web [34–35]. Temperature also decreases at higher latitudes, thus increasing the mixing of CO_2_ (^13^C-depleted) in sea water and diminishing δ^13^C at the base of the food web [33,36]. In order to assess pinniped foraging habits, neonate stable isotopes are commonly used, because at this age, pups are completely milk-dependent. This makes them effective indicators for their mothers, who catabolize their tissues to produce milk, emulating a predator-prey relationship [16,37].

This study provides data regarding GFS and CSL abundance on the SBA throughout 2014 and 2015 during ENSO conditions. We also assessed foraging habits of both species via stable isotope (N and C) analysis of pup fur, using samples retrieved during the breeding seasons (summer) of both years and we discuss our findings in the context of recent climate variation. Our contribution constitutes evidence that supports the role of these marine mammals as indicators of change in the ecosystem.

## Materials and Methods

The fieldwork was carried out with permits SGPA/DGVS/11744/13 and SGPA/ DGVS/00195/15 under Secretaría de Medio Ambiente y Recursos Naturales (SEMARNAT) through Dirección General de Vida Silvestre (DGVS). These study-specific permits and the Institutional Animal Care and Use Committee (IACUC) from Facultad de Ciencias Naturales at Universidad Autónoma de Querétaro (UAQ), approved all collection methods used for this work. Additionally, two of the authors, GS-R and KA-W, have veterinarian formations (Professional Licenses 8111223 and 3275601, respectively).

**Fig 1:**
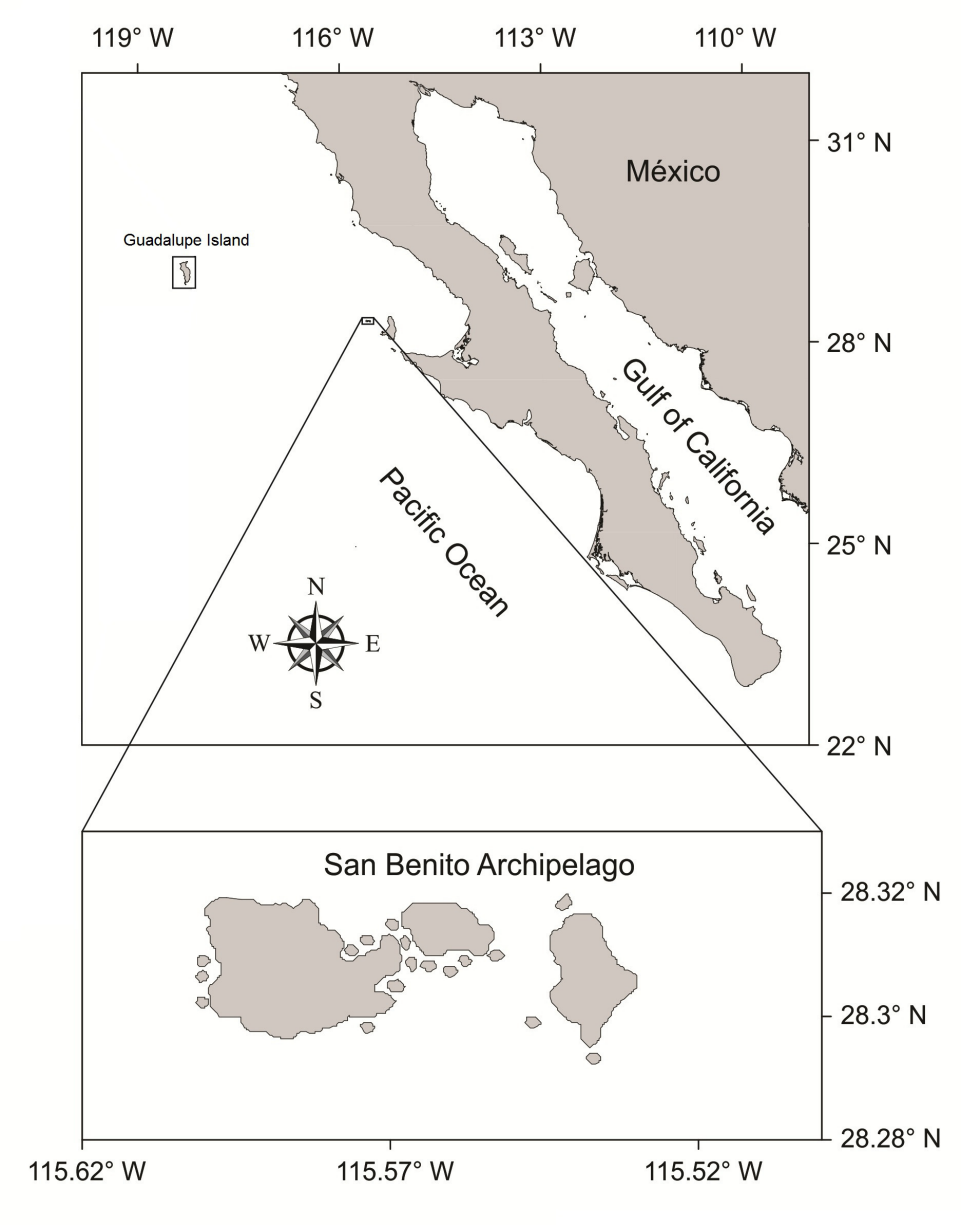
Location of the San Benito Archipelago on the west coast of the Baja California Peninsula, Mexico.

Sampling was conducted at the SBA (6.4 km^2^; 28°18’N and 115°34’W), a group of three islands (East, Middle, and West) located 270 km southeast of GI and 75 km northwest of the Baja California Peninsula, Mexico (Fig 1).

### Guadalupe fur seal and California sea lion abundance (2014–2015)

We conducted nine GFS surveys during 2014 (4–13 February; 30 April—5 May; 30 June—6 July; 11–14 September; 5–9 December) and 2015 (17–24 February; 4–12 May; 30 June—6 July; 14–22 September).

Counts were performed always by the same person and under weather conditions as similar as possible, using one of two techniques commonly employed in pinniped surveys [8,10,38,39]. A walking survey was performed once per trip on the Middle and West Islands, approaching colonies with caution in order to avoid disturbance. At the East Island, where cliffs are too steep to access on foot, we conducted one survey per trip, counting from a boat at an average distance of 30 m. Each site was surveyed using the same technique during each excursion in order to ensure data consistency. These two different approaches were complementary; each maximized the survey efficiency for the specific location in question, guaranteeing we recorded the largest number of animals possible at each location [10]. Because of the large variation in substrate along islands and between the two species, we did not apply correction factors; all temporal data were compared under the same conditions [9,10].

We grouped GFSs and CSLs into five age- and sex-classes: pups (P), immature non-pups (I), adult females (AF), adult males (AM), and miscellaneous (M) (all animals not fully identified) [8,10,38]. We used chi-square and Z tests to determine differences between the observed proportions.

### Guadalupe fur seal and California sea lion foraging habits (δ^15^N and δ^13^C)

Sampled pups (morphometrics and fur) were handled for short periods of time (2–3 min each) in order to diminish stress [16,37]. No individual was harmed during the development of this study.

During part of the breeding season (July), we captured and immobilized most of GFS pups (19 in 2014; 16 in 2015) and 30 CSL pups for each year. The number of GFS pups captured was low as few births occur at the SBA [9,10]. After weighing each pup using a hanging scale with a 25 kg capacity and measuring the standard length (distance between nose and tail), we cut a 2 x 3 cm fur sample from the dorsal region. Samples were stored in paper envelopes labeled with pertinent identifying information.

All fur samples were processed in the Chemistry Laboratory at the Centro Interdisciplinario de Ciencias Marinas (CICIMAR; Interdisciplinary Center for Marine Sciences) of the Instituto Politécnico Nacional (IPN; National Polytechnic Institute). Samples were washed with distilled water and chloroform/methanol (1:1) to remove impurities. An agate mortar was used to homogenize the samples and 0.8–1.2 mg were weighed on an analytical microbalance to a 0.001 mg precision. The samples were stored in 8 x 5 mm tin capsules and sent to the Earth and Planetary Sciences Stable Isotope Laboratory at the University of California (UCSC), where the isotopic (δ^15^N and δ^13^C values) determination was made using a Carlo Erba 1108 elemental analyzer coupled to a ThermoFinnigan Delta Plus XP isotope ratio mass spectrometer with an analytical precision of ±0.2‰ for both stable isotopes. For both C and N, the proportion of stable isotopes is represented using delta (δ) and calculated based on the following equation [40]:

$$\delta^{15}N\;or\;\delta^{13}\text{C}=1000[\text{R}\;\text{sample}/\text{R}\;\text{standard}-1]$$

Where *R* is the ratio of ^15^N/^14^N or ^13^C/^12^C for the sample and the standard, respectively. The elemental composition of C and N was estimated based on standards of known values: Vienna Pee Dee Belemnite (δ^13^C *=* 0.011*‰*) and atmospheric N_2_ (δ^15^N = 0.004‰).

The Bayesian method SIBER (Stable Isotope Bayesian Ellipse in R) was used to determine the niche area using the SIAR (Stable Isotope Analysis in R) 4. 2 package in R. This analysis is complementary to the polygon area method of determining niche area and involves measurements based on ellipses, which are not influenced by sample size or outliers, as is the case with polygon and convex hull techniques, which involve all values. We used Monte Carlo simulations to form the bivariate ellipses (^15^N and ^13^C), surrounding the data points in the 95% confidence interval for the distributions of both stable isotopes [41]. Further t-test comparisons were performed to search for differences between paired groups.

### Environmental variability (2014–2015)

Based on the fact that 2015 was declared as an ENSO year [24], we examined two of the main variables responsible for shaping the marine mammal´s distribution patterns [42]; sea surface temperature (SST) and chlorophyll-a (Chl-a) concentration, which is a proxy of primary productivity [43–44]. We obtained data for these variables from MODIS Aqua satellite images, with a spatial resolution of 9 x 9 km. These are available from NASA’s GIOVANNI website (http://giovanni.gsfc.nasa.gov/giovanni/). We converted both variables to ASCII format using ArcGIS software and calculated the mean values of SST and Chl-a within satellite images, corresponded to ~70 days prior to July of 2014 and 2015, when the highest abundances were recorded for both otariids and when fur samples were sampled. This amount of days is the approximate period of isotopic information that fur represents [16,37].

## Results

### Guadalupe fur seal abundance (2014–2015)

Differences in the interannual abundance of GFSs were observed, particularly during May, July, and September, when the 2015 values were nearly half of those recorded during the same months in 2014. The GFS reached its peak in July (2014 and 2015); however, in 2015 the census evidenced a decline of 59.7% of the total abundance and of 42.9% of pups, relative to 2014 (Table 1).

**Table 1:** Abundance of Guadalupe fur seals (*Arctocephalus philippii townsendi*) at the San Benito Archipelago during 2014 and most of 2015.

Guadalupe fur seals	February		May		July		September		December	
	2014	2015	2014	2015	2014	2015	2014	2015	2014	2015
Adult males	0	0	0	0	2	5	0	0	0	-
Adult females	7	5	2	0	6	13	0	5	5	-
Immatures	13	30	2,089	1,217	3,674	1,460	1,408	791	487	-
Pups	14	15	4	3	28	16	25	5	27	-
Total	34	50	2,095	1,220	3,710	1,494	1,433	801	519	-

**Fig 2:**
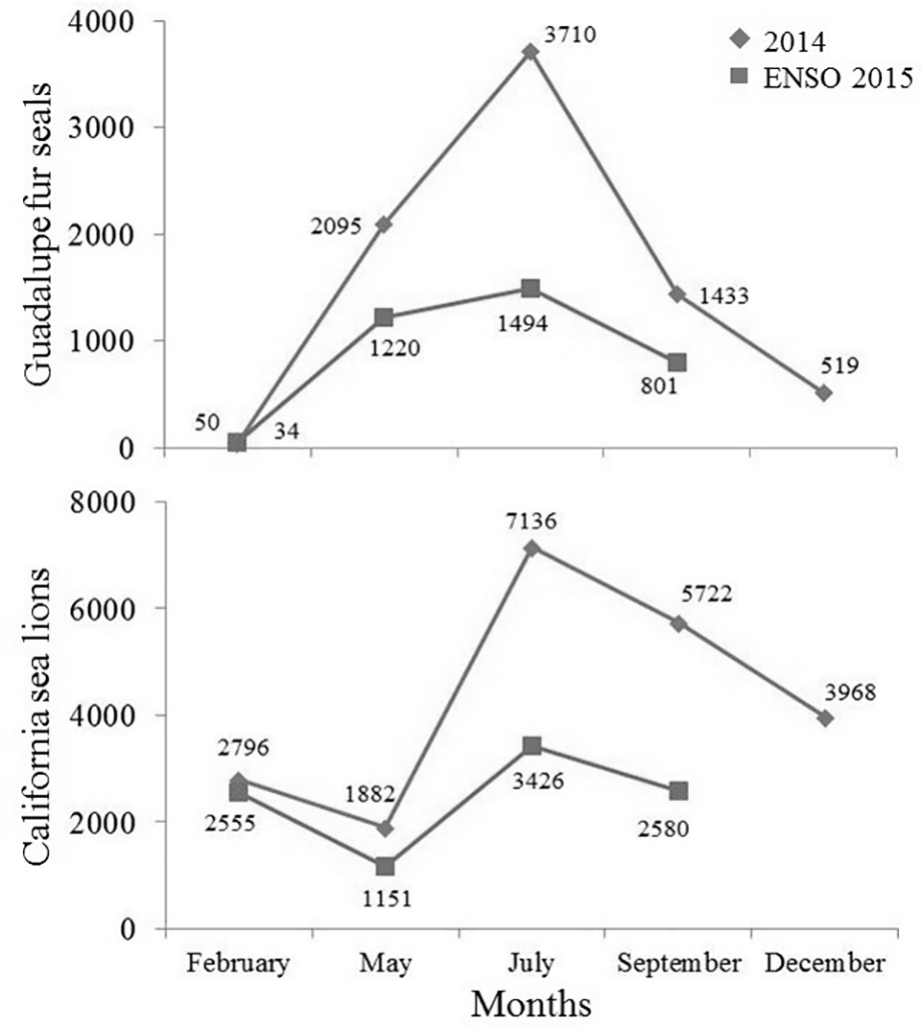
Overall monthly abundance of Guadalupe fur seals (*Arctocephalus philippii townsendi*) and California sea lions (*Zalophus californianus*) at the San Benito Archipelago, throughout 2014 and 2015.

In both 2014 (X^2^ = 66.8, df = 4, P = 0.00001) and 2015 (X^2^ = 37.3, df = 3, P = 0.00001) GFSs resided seasonally at the SBA (Fig 2). The lowest abundance occurred in February (34 in 2014; 50 in 2015); the peak was in July (3,710 in 2014; 1,494 in 2015). Immature individuals made up the majority of the colony, particularly from May to December when they comprised around 95%. Both female and male adults were scarce, with their highest proportions occurring in February (20.5% in 2014; 10% in 2015), while during the rest of the year they represented from 0 to 1.2% of the colony. As observed for adults, pups showed the highest proportions in February (41.2% in 2014; 30% in 2015); however, pups were most common during breeding seasons (28 in 2014; 16 in 2015) (Table 1). No significant differences in GFS pup proportions were found within the colony, between summer of 2014 (0.75%) and 2015 (1.1%) (Z = 0.3, P = 0.796).

### California sea lion abundance (2014–2015)

As with GFSs, CSL abundance varied between 2014 and 2015, particularly in May, July, and September, with 2015 values being roughly half of those observed in 2014 (Table 2). Relative to GFSs, CSLs had a more stable abundance within each year. Their peak abundance was maintained during the breeding season in July (7,136 in 2014; 3,426 in 2015), decreasing gradually until May when the lowest abundance was recorded (1,882 in 2014; 1,151 in 2015) (Fig 2). The dominant categories were adult females (up to 40% in July 2014) and pups (from 32% in July 2015 to 63.7% in December 2014), with proportions varying by month and year of survey. Adult males were rarely present (0 to 6 individuals) throughout the year, except during the breeding season in July, when they were most abundant (411 in 2014; 197 in 2015).

**Table 2:** Abundance of California sea lions (*Zalophus californianus*) at the San Benito Archipelago during 2014 and most of 2015.

California sea	February		May		July		September		December		
lions	2014	2015	2014	2015	2014	2015	2014	2015	2014	2015	
Adult males	6	2	2	0	411	197	3	0	0		-
Adult females	1,086	761	729	345	2,861	1,261	1,437	888	1,080		-
Immatures	151	67	102	69	659	744	336	280	197		-
Pups	1,373	1,567	979	609	2,902	1,112	3,538	1,297	2,531		-
Miscellaneous	180	158	70	128	303	112	408	115	160		-
Total	2,796	2,555	1,882	1,151	7,136	3,426	5,722	2,580	3,968		-

Although CSLs peaked during July both years; individuals of all classes decreased by 52.0% in 2015. This trend was also observed for pups, which declined 61.7%, relative to July 2014. In May 2015, the whole colony- and pup abundance decreased 38.9% and 37.8, respectively, relative to May 2014. Meanwhile, in September 2015, whole colony and pup abundance declined 54.9% and 63.4%, relative to September 2014 (Table 2). No significant differences in CSL pup proportions were found within the colony, between the 2014 (40.6%) and 2015 (32.6%) breeding seasons (Z = 1.2, P = 0.240).

### Mass and lengths for Guadalupe fur seal and California sea lion pups sampled for isotopic assessment (breeding seasons 2014–2015)

The mean (±SD) mass of GFS pups was 7.7 ± 0.9 kg, and the mean length (±SD) was 71.5 ± 4.6 cm in 2014 (n = 19). In 2015 (n = 14), values were 7.8 ± 1.6 kg and 69.5 ± 4.3 cm, respectively. There were no significant differences in either measurement between the two years (T-test, P > 0.05). In contrast, mean (±SD) weight of CSL pups was 9.9 ± 1.8 kg in 2014 (n = 30), which was statistically higher (t = 2.9, P = 0.004) than the mean weight recorded in 2015 (n = 30) 8.7 ± 1.5 kg. Mean pup length did not vary between years (t = 0.7, P > 0.05) (2014 = 79.5 ± 4.1 cm; 2015 = 78.8± 4.1 cm).

### Stable isotope analysis for both otariid pups (2014–2015)

**Fig 3:**
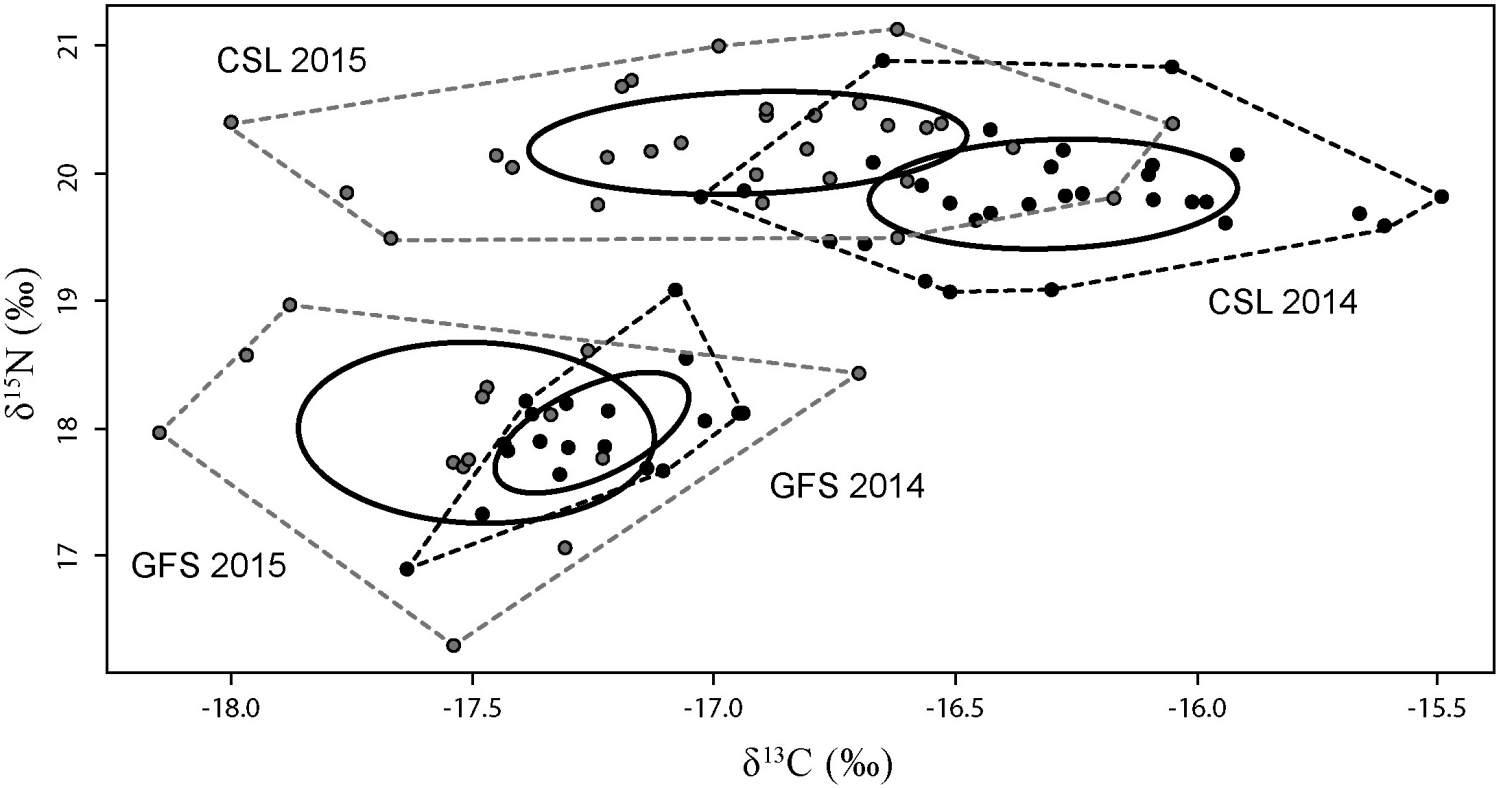
Isotopic niche (δ^13^C- δ^15^N) of Guadalupe fur seals (*Arctocephalus philippii townsendi*) and California sea lions (*Zalophus californianus*) at the San Benito Archipelago during spring-summer of 2014 and 2015. Black circles represent 2014 for both species; while grey circles represent 2015.

Regardless of the survey year, CSL pups had consistently higher isotope values (δ^13^C_2014_: t = 10.3, P = 0.00001; δ^15^N_2014_: t = 14.9, P = 0.00001; δ^13^C_2015_: *t* = 4.1, P = 0.0001; δ^15^N_2015_: t = 13.9, P = 0.00001) than GFS pups. There was no overlap between the isotopic niches of the two otariids (Fig 3).

### Guadalupe fur seal foraging habits

In 2014, mean (±SD) isotopic values for GFSs pups were -17.2 ± 0.2‰ (δ^13^C) and 17.9 ± 0.4‰ (δ^15^N), while the total isotopic niche areas were 0.6 (polygon) and 0.2 (ellipse). In 2015, the mean (±SD) values were -17.5 ± 0.3‰ (δ^13^C) and 17.8 ± 0.7‰ (δ^15^N), with total isotopic niche areas of 2.0 (polygon) and 0.8 (ellipse). We identified significant differences in δ^13^C (t = 2.5, P = 0.02), but not δ^15^N (t = 0.04, P > 0.05) between years. In 2014, 58% (n = 11) of GFSs were located within the 2015 GFS ellipse (Fig 3).

### California sea lion foraging habits

In 2014, mean (±SD) CSL pup values were -16.2 ± 0.4‰ (δ^13^C) and 19.8 ± 0.4‰ (δ^15^N). The polygon and ellipse isotopic niche areas were 1.8 and 0.5, respectively. In 2015, the mean (±SD) values were -16.9 ± 0.4‰ (δ^13^C) and 20.2 ± 0.4‰ (δ^15^N), while the isotopic niche areas were 2.2 (polygon) and 0.6 (ellipse). We identified significant differences between years, for both stable isotopes (δ^13^C: t = 3.8, P = 0.00001; δ^15^N: t = -5.9, P = 0.0003). In 2014, only two CSLs were located within the 2015 CSL ellipse (Fig 3).

### Environmental variability (2014–2015)

**Fig 4:**
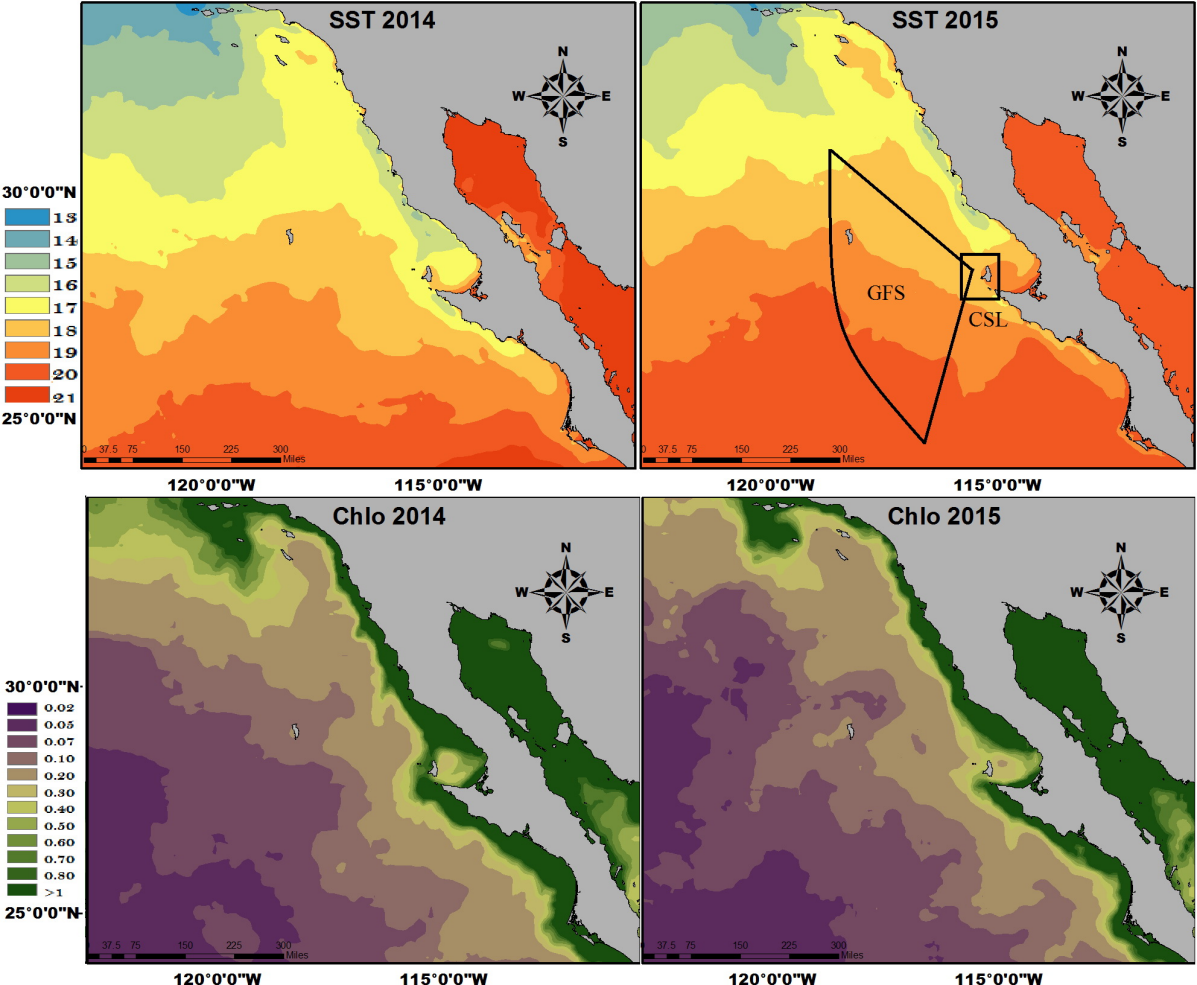
Mean sea surface temperature (°C) and chlorophyll (mg/m^3^) adjacent to the Baja California Peninsula, during 70 days prior to July 2014 and 2015. Hypothetical foraging ranges of Guadalupe fur seals (GFS) and California sea lions (CSL) were traced in the upper right map.

Mean SST adjacent to the Baja California Peninsula, ~70 days prior to July 2014 and 2015, when the highest abundances of GFSs and CSLs were recorded and when fur samples for stable isotope analysis were collected, increased 2°C in 2015 compared to 2014. Chl-a concentration was lower in 2015 (0.2–1 mg/m^3^) than in 2014 (0.4–1 mg/m^3^) (Fig 4).

## Discussion

This study provides important ecological information on GFS and CSL abundance and foraging on the SBA during two consecutive years, one of which (2015) was characterized by intense ENSO conditions during most of the year [24]. Both species decreased significantly in abundance during 2015. This finding, coupled with isotopic niche changes, highlights the importance of these predators as sentinel species for environmental anomalies, such as warming and decline of primary productivity. SST adjacent to the Baja California Peninsula in 2014 and 2015 (May-July for both years), was 2–3°C higher, relative to 2012 and 2013 on the same dates; while Chl-a of 2014 and 2015 was lower than 2012 and 2013, when a concentration of 1–10 mg/m^3^ was recorded (http://giovanni.gsfc.nasa.gov/giovanni/). The presence of ENSO conditions, exacerbated by warm conditions from “The Blob” in 2014, may have modified prey availability in 2015 and thus decreased the amount of time animals spend on land, due to increased foraging effort and a subsequent potential impact on reproductive success [21].

### Abundance of Guadalupe fur seals on the San Benito Archipelago

The 3,710 GFSs counted in July 2014 supports the increasing long-term trend reported for the SBA colony [11]. This trend is more evident during the summer. For instance, 582 individuals were counted in summer 2000 compared to 4,572 in summer 2012 [9,10]. However their abundance declined in July 2014 and in July 2015 reached its lowest (1,494) since 2006. A significant decline occurred between 2014 and 2015 (59.7%) for all classes and 42.9% for pups. Such interannual variation could be explained by the seasonal variation in GFS occupation of the SBA, a fact that has been determined previously, with the greatest abundance occurring during summer 2012 (4,572) and the lowest in winter 2013 (58) [9]. However, our study is the first to describe the seasonality of GFS occupation at the SBA using multiple surveys over the course of two years, with significantly different abundances observed during July and February, as well as a clear transition throughout the year.

The seasonal presence of GFSs at the SBA involves primarily immature individuals that migrate from GI [10]. In pinnipeds, this type of recolonization is the result of the species’ dispersion, the availability of suitable habitats, and subsequent reproductive success [45]. The process is gradual and is related to the arrival of young males to a resting area with a high abundance of immature animals [46,47] such as the SBA. The only month during which the proportion of adult females and pups exceeded, or was closer to, that of immature animals was February of both years, when nearly all GFSs had abandoned the SBA, making the reduced abundance of mothers and neonate pups (28 in 2014; 16 in 2015) on the SBA more evident.

Migration of GFSs between GI and the SBA could help explain the abrupt reduction of GFSs at the SBA during the 2015 ENSO. We propose that GFSs move to different -potentially more distant- foraging areas, as has been reported for other otariids during ENSO events [21]. During the 2015 ENSO, GFSs were sighted in areas of Mexico (La Paz Bay, Gulf of California) with no previous records of the species [48] and an unprecedented mortality event (~80 GFSs) was reported in California, USA, between January and August 2015 [49]. These unusual events, together with isotopic evidence, support the hypothesis of a wider dispersion of GFSs in 2015 relative to 2014.

The recent annual (summer) decline of the GFS colony at SBA (4,572 in 2012, 1,969 in 2013, 3,710 in 2014), and especially in 2015 (1,494), addresses the necessity of more surveys, in order to clarify abundance patterns, which no longer shows an increase trend of years past, at least since 2013. This time window coincides with the recent increase of SST in Northeast Pacific [23]. Similar studies should also be undertaken at GI, which is the species’ only established breeding colony.

### Foraging habits of the Guadalupe fur seals from the San Benito Archipelago

During both years, GFSs had lower δ^13^C and δ^15^N values than CSLs. This trend can be explained by the more oceanic foraging (δ^13^C) and lower trophic position (δ^15^N) of GFSs [50].

Only the δ^13^C values were significantly different for GFSs between 2014 and 2015; however, the difference was small (0.3‰) and thus should be considered with caution. The interannual isotopic difference could also be also explained by the 2015 isotopic niche breadth, which was more than three times that in 2014. The greater dispersion in δ^13^C may be related to the use of a larger number of foraging areas with distinct isotopic base values [51], as a result of longer trips undertaken during ENSO events, as has been reported in other species like the South American (*Arctocephalus australis*) and Galapagos fur seals (*A*. *galapagoensis*) [52,53]. Extreme displacement has also been reported for GFSs in California and Mexico during the 2015 ENSO [48,54]. The possibility of more oceanic foraging trips in 2015 is supported by our more negative values for δ^13^C, as the baseline values for this stable isotope decrease with distance from the coast [27–28]. We cannot rule out a foraging displacement toward a higher latitude in 2015 (^13^C-depleted baseline), as has been described for multiple pinniped species and their foraging movements [51,55]. However, this would only explain the variation of δ^13^C; it was not observed for δ^15^N, which also tends to decline with increasing latitude [32,55].

The few GFSs pups born at the SBA did not show significant differences in weight or length between the two years, and these were even higher or similar (7.3 kg and 70.5 cm) than a year before (2013) our study, on the same days [11]. This may indicate that the observed changes in δ^13^C did not exert a negative effect on pups, at least during the first month of life. This may be explained by the wide dispersal capacity (~600 km) observed during GFS foraging trips [13], increasing the possibility of taking advantage of a wider offshore foraging ground, which is typical of the species [18]; this behavior would be opposite to a restricted dispersal, with few foraging options. Additionally, GFS pup proportions within the SBA was not different between July 2014 and 2015; although a slightly higher proportion was observed in 2015. Such a phenomenon should be assessed over time, particularly during the anomalous environmental conditions that have caused recent unusual mortality events in immature GFSs in California [49].

### Abundance of California sea lions on the San Benito Archipelago

The abundance of CSLs varied by season during both years, as previously observed at the SBA in 2014 [8]. The greatest abundance of individuals was observed during the breeding season in July, when many adult animals are found on land for reproduction and a large number of neonates are born. As a result, more CSLs are counted during this period than during other months. In July 2013, a similar survey counted 8,859 CSLs [8], which is higher than the abundance observed during our July 2014 survey (7,136). However, historic CSL counts at the SBA suggest (prior to 2015) colony stability, and the decrease between 2013 and 2014 may have been related to a change in prey availability caused by the large mass of warm water known as “The Blob,” which emerged in the North Pacific at the end of 2013 and had effect on the coastal waters where CSL forage [8,22,23]. The 2015 ENSO caused an additional SST increase, and Chl-a decrease, relative to 2014. Thus, the decrease in CSL numbers at the SBA now involves a two-year phenomenon (2014–2015). The increase in the SST and decline in primary productivity likely diminished prey availability, forcing female CSLs to increase their foraging efforts, as has been reported for the same species in California, USA [56], and Magdalena Bay, Mexico, during the 1982–1983 ENSO. Increased foraging effort causes a decrease in the number of individuals on land and has a negative effect on pup body condition and survival [57]. This was evidenced in the present study, and has been recorded for other otariids, such as the Galapagos (*Zalophus wollebaeki*) and South American (*Otaria flavescens*) sea lions during the 1982–1983 and 1997–1998 ENSOs [52,58]. In our work, 2015 pup production was nearly three times lower than 2014. In July 2013, a total of 3,944 CSL pups were counted [8] (S1 File), reflecting a decrease in the number of births or pup survival on the SBA during the gradual (2013–2015) warming of the area, from 26% in 2014 to 72% in 2015. It is imperative to continue these surveys over time, in order to better understand the effect of climate change on these rookeries.

### Foraging habits of the California sea lions from the San Benito Archipelago

In 2015, CSL δ^13^C values were lower, while δ^15^N were higher relative to 2014, denoting a possible variation in foraging areas or in types of prey being consumed. This change in both stable isotopes for CSLs, in contrast to the pattern observed for GFSs, who only showed significant differences in δ^13^C, may be the result of a more marked change in foraging patterns of CSLs and the subsequent lower body mass of their pups in 2015. The increased CSL sensitivity to environmental variation around the SBA and the negative effect on pups may be related to the presence of CSL adult females on this archipelago throughout the year [8], as well as the fact that this species makes shorter foraging trips than GFSs [13,17]. Female CSLs exploit resources within a radius of ≤100 km [59], returning to feed their young following foraging trips that last up to three days [19]; in contrast, female GFSs foraging trips last up to 14 days [60]. This difference in dispersal between both species should imply a higher foraging limitation for CSLs during anomalous conditions.

A telemetry study of adult female CSLs in California, USA, revealed that the greatest movements away from their rookery occurred during unusual warming events (2004–2005), implying an increase in foraging effort [61]. Longer and more oceanic CSL incursions could explain our more negative δ^13^C values, as well as the three times larger isotopic niche breadth found during the 2015 ENSO. We cannot rule out movements toward higher latitudes with depleted basal signals; however, as with GFSs, this latitudinal effect would only explain the lower δ^13^C observed. Scat analysis should be performed to better interpret the increase in δ^15^N in 2015; however, previous studies have reported ^15^N-enriched zooplankton, at the base of the trophic web during ENSO events [62], perhaps due to the tropicalization of the environment and the increased effect of denitrifying bacteria, whose processes increase the δ^15^N from the base [35] to apex predators [51]. It has also been hypothesized that nutritional stress contributes to elevated δ^15^N values in tissues. Fasting can cause a negative nitrogen balance and a subsequent ^15^N-enriched nitrogen pool, which is involved in amino acid synthesis [63–64].

## Final Remarks

Both species suffered declines of abundance at the SBA during the 2015 ENSO, as well as isotopic niche changes, with CSLs being more impacted than GFSs, which maybe more resilient to climate change because of greater foraging range. These abrupt declines of both otariids were probably amplified by “The Blob” phenomenon of 2014. This finding is plausibly related to longer foraging trips by the two species. However, without being able to tag individual animals, we cannot rule out that the decline in abundance is related to higher mortality levels or redistribution. Moreover, compared to 2014, we observed fewer pups of both species during 2015, most likely due to reduced female fecundity, lower pup survival, or both. Once more, we cannot distinguish between these two alternatives because the pups were not tagged, and could not be tracked over time. Comparative analysis of pup health and immune parameters between years should help determine whether pups were more likely to die in 2015 due to hampered health.

Pup fur isotope values showed differences in the foraging niches used by the two species, two to three months prior to sampling (July); thus, they are only representative of part of the year. Future studies should increase the time frame examined here in order to better assess patterns throughout the year. The main hypothesis of this study is that both otariids undertook more oceanic foraging trips during the 2015 ENSO. However, telemetry studies should be conducted in order to test this hypothesis.

It is imperative to continue these surveys over time; for instance, in February 2016, during a survey that was not part of this study, we recorded a decline of ~80% of the total CSL abundance at the SBA, compared to the numbers observed for the same month in 2014 and 2015, which were similar. A large number of emaciated pups was also recorded in February 2016, as well as a ~77% decline relative to the same dates in 2014 and 2015.

The present study provides evidence on how oceanographic anomalies impact marine mammal populations, modifying their foraging areas and exerting a negative effect on pup body condition and/or survival, as has previously been observed among CSLs in southern California during the last three years [56]. Moreover, adverse effects that alter GFS prey availability [48–49] may be an obstacle to their recovery. Thus, environmental factors should be considered when determining their conservation status. Our results for both otariids during the 2015 ENSO highlight the importance of these species as sentinels of their surrounding ecosystem.

## Supporting Information

S1 File Abundance of California sea lions (*Zalophus californianus*) (CSLs) and Guadalupe fur seals (*Arctocephalus townsendi*) (GFSs) at San Benito. Information from 2012 and 2013 are included for the CLSs.
(XLSX)
